# Activation of eNOS by D-pinitol Induces an Endothelium-Dependent Vasodilatation in Mouse Mesenteric Artery

**DOI:** 10.3389/fphar.2018.00528

**Published:** 2018-05-22

**Authors:** Luciana N. Moreira, Josiane F. Silva, Grazielle C. Silva, Virgínia S. Lemos, Steyner F. Cortes

**Affiliations:** ^1^Department of Pharmacology, Institute of Biological Sciences, Universidade Federal de Minas Gerais, Belo Horizonte, Brazil; ^2^Department of Physiology and Biophysics, Universidade Federal de Minas Gerais, Belo Horizonte, Brazil

**Keywords:** calcium-calmodulin complex, D-pinitol, endothelium, mesenteric artery, nitric oxide synthase

## Abstract

D-pinitol is a cyclitol present in several edible plant species and extensively investigated for the treatment of metabolic diseases in humans, as food supplement, and demonstrated protective effects in the cardiovascular system. For these reasons, the present work aimed at investigating the mechanisms involved in the vascular effects of D-pinitol in mouse mesenteric artery. Mesenteric arteries from male C57BL/6 mice were mounted in a wire myograph. Nitrite was measured by the 2,3-diaminonaphthalene (DAN) method. Protein expression and phosphorylation were measured by Western blot. The systolic blood pressure (SBP) was measured by tail-cuff plethysmography. D-pinitol induced a concentration-dependent vasodilatation in endothelium-intact, but not in endothelium-denuded arteries. Nω-Nitro-L-arginine methyl ester (300 μM) abolished the effect of D-pinitol, while 1H-[1,2,4]oxadiazolo[4,3-a]quinoxalin-1-one (ODQ; 10 μM) shifted the concentration-response curve to the right. KN-93 (1 μM) blunted the vasodilator effect of D-pinitol, but H-89 (0.1 μM) did not change it. 1-[2-(Trifluoromethyl) phenyl]imidazole (300 μM), indomethacin (10 μM), celecoxib (5 μM), wortmannin (1 μM), ruthenium red (10 μM), tiron (10 μM), MnTMPyP (30 μM), MPP (0.1 μM), PHTPP (0.1 μM), and atropine (1 μM) did not change the effect of D-pinitol. D-pinitol increased the concentration of nitrite, which was inhibited by L-NAME and calmidazolium (10 μM). D-pinitol increased the phosphorylation level of eNOS activation site at Ser^1177^ and reduced the phosphorylation level of its inactivation site at Thr^495^. In normotensive mice, the intraperitoneal administration of D-pinitol (10 mg/kg) induced a significant reduction of the SBP after 30 min. The present results led us to conclude that D-pinitol has an endothelium- and NO-dependent vasodilator effect in mouse mesenteric artery through a mechanism dependent on the activation of eNOS by the calcium-calmodulin complex, which can explain its hypotensive effect in mice.

## Introduction

Inositols are cyclohexane polyols also known as cyclitols. They are arranged in nine stereoisomers: scyllo, myo, neo, chiro, cis, mucus, and allo (Thomas et al., 2016). Inositols play an important role in cell physiology as precursors of second messengers involved in the secretory processes, metabolism, contraction, and proliferation (Thomas et al., 2016). Moreover, inositols have some clinical uses in the treatment of polycystic ovary syndrome and have been considered as an option as a food supplement for treatment or prevention of gestational and type 2 diabetes mellitus (Facchinetti et al., 2015; Celentano et al., 2016). The improvement in the metabolic parameters observed after the use of inositols in polycystic ovary syndrome, and type 2 diabetes mellitus suggests that they may have a protective effect on the cardiovascular system (Cussons et al., 2006).

D-pinitol (3-*O*-methyl-D-chiro-inositol) is a cyclitol present in several edible plants, including soybean and carob (Kawai and Kumazawa, 1982; Baumgartner et al., 1986). The chemical similarity suggests that pinitol is a natural source of D-chiro-inositol *in vivo* (Davis et al., 2000). Pinitol has been described as an antidiabetic drug, with insulin-like effect in an animal model of diabetes (Bates et al., 2000), and the ability to potentiate the activity of insulin through the translocation of glucose transporter 4 in skeletal muscle of mice (Dang et al., 2010). In humans with type 2 diabetes mellitus, treatment with pinitol improved the glycemic control, reduced the adipocytokine level, and reduced the metabolic parameters associated with cardiovascular risk (Kim et al., 2005, 2012). In the cardiovascular system, D-pinitol was shown to prevent the endothelial dysfunction induced by diabetes in rat aorta and mesenteric arteries (Nascimento et al., 2006). This protective effect was attributed to its antioxidant effect, which seemed to be responsible for the preservation of nitric oxide (NO) signaling. It is noteworthy that the polycystic ovary syndrome is also associated with an endothelium dysfunction (Paradisi et al., 2001) and the treatment with cyclitols may also have a protective effect in this disease (Croze and Soulage, 2013).

D-pinitol has been extensively investigated for the treatment of metabolic diseases in humans, and as a food supplement, for these reasons, a more detailed investigation of the vascular effect of this cyclitol is necessary. Therefore, the present work aimed at investigating the vascular effect of D-pinitol in small mesenteric arteries from mice.

## Materials and Methods

### Animals

Sixty-six male C57BL/6 mice, aged 10–12 weeks, were used in the present study. The experiments were performed in accordance with the recommendations of the ethics committee of the Universidade Federal de Minas Gerais. The experimental protocols were approved by the Animal Ethics Committee (protocol 170/2014).

### Vascular Reactivity

Mice were euthanized by decapitation, the abdomen was cut, and the mesenteric bed was quickly removed and placed in a dissecting plate with physiological salt solution (PSS) with the following composition (mM): NaCl 119.0; KCl 4.7; KH_2_PO_4_ 0.4; NaHCO_3_ 14.9; MgSO_4_.7H_2_O 1.17; CaCl_2_.2H_2_O 2.5; and glucose 5.5. A segment of the second branch of the mesenteric artery was dissected, and the adipose and connective tissues were removed. The arteries were sectioned into rings (1.6–2.0 mm long) with an internal diameter ranging from 150 to 250 μm. The rings were mounted in a wire myograph (620M, DMT, Denmark), kept in carbogen aerated PSS at 37°C. After mounting, the artery was stretched to a length that yielded a circumference equivalent to 90% of that given by an internal pressure of 100 mmHg; this required a load of approximately 200 mg. The vessel was maintained for an equilibration period of 60 min. The mechanical activity was recorded isometrically as previously described (Silva et al., 2016). The functionality of the arteries was observed by the contraction induced by phenylephrine (3 μM) and by the vasodilator effect induced by acetylcholine (ACh, 10 μM) in arteries pre-contracted with phenylephrine. Arteries with ACh-induced vasodilatation higher than 70% were considered with functional endothelium. In some experimental procedures, the endothelium was removed by rubbing the lumen slightly with the tungsten wire. The removal of the endothelium was confirmed by the absence of vasodilatation induced by ACh in precontracted arteries. The vasodilator effect of D-pinitol was evaluated by concentration-response curves (1 nM to 100 μM) in mesenteric arteries in the presence and the absence of a functional endothelium pre-contracted with phenylephrine (3 μM). The participation of nitric oxide synthase (NOS) was investigated in arteries pretreated with Nω-nitro-L-arginine-methyl-ester (L-NAME; 300 μM), a non-selective inhibitor of NOS, and 1-(2-trifluoromethylphenyl) imidazole (TRIM; 300 μM), a selective inhibitor of neuronal NOS (nNOS). The activation of guanylate cyclase was investigated with 1H- [1,2,4]-oxadiazolo[4,3-a]quinoxalin-1-one (ODQ; 10 μM). The involvement of cyclooxygenase (COX) 1 and 2, phosphatidylinositol-3-kinase (PI3K), Ca^2+^/calmodulin-dependent kinase II (CaMKII), and non-selective cationic channels was verified in arteries pretreated with indomethacin (10 μM), celecoxib (5 μM), wortmannin (1 μM), KN-93 (1 μM), and ruthenium red (10 μM), respectively. Tiron (10 μM) and MnTMPyP (30 μM), a cell-permeable analog of superoxide dismutase, were used to investigate the action of antioxidant drugs on the vasodilator effect of D-pinitol. The participation of muscarinic receptors and α and β estrogen receptors was investigated in arteries pretreated with atropine (1 μM), MPP (0.1 μM), and PHTPP (0.1 μM), respectively.

### Nitrite Measurement in Mouse Mesenteric Artery

The assessment of NO production in the mesenteric artery was performed indirectly by the measurement of nitrite (NO_2_^-^) using the fluorescence method with 2,3-diaminonaphthalene (DAN), according to Silva et al. (2016). The mesenteric artery branches were placed in PSS, at 37°C in 5% CO_2_ atmosphere. Samples were collected in the absence (basal) or the presence of D-pinitol (20 μM) or ACh (10 μM). The involvement of NOS and calmodulin in the production of nitrite was evaluated in the presence of L-NAME (300 μM) and calmidazolium (10 μM), respectively. 150 μl samples were collected, added to 150 μl of purified water, followed by the immediate addition of 15 μl fresh DAN solution (0.05 mg/l in 0.62 M HCl) in 96-well opaque black plates (Costar^®^, United States). The reaction proceeded for 10 min at room temperature and protected from light. After this period, the reaction was stopped with 5 μl of NaOH (2.8 N) and the absorbance determined using a spectrofluorometer (Fluoroskan Ascent FL, Thermo Scientific) at 365 and 415 nm, as respective excitation and emission wavelengths. The nitrite concentration in the samples was calculated using a standard curve with predetermined concentrations of sodium nitrite in each experiment and normalized by the amount of protein in the branches. The results were expressed in [nitrite] nM/μg of protein.

### Western Blot Analysis of eNOS Phosphorylation

Western blot analysis was performed as previously described (Silva et al., 2016), with some modifications. Briefly, a pool of mesenteric resistance arteries from six animals was stabilized in PSS aerated with a carbogenic mixture (95% O_2_ and 5% CO_2_) at 37°C. D-pinitol (20 μM) was added and after 5, 15, or 30 min, the arteries were collected, and frozen at -80°C. Arteries that were not stimulated by D-pinitol were used to determine the basal level of phosphorylation (time zero). After, the tissues were homogenized in the presence of lysis buffer (150 mM NaCl; 50 mM Tris; 5 mM EDTA.2Na; and 1 mM MgCl_2_) plus 0.3% Triton X-100, 0.5% SDS, and protease inhibitors cocktail (Sigma Fast^®^, Sigma), supplemented with a cocktail of protease inhibitors (20 mM NaF, 0.1 mM Na_3_VO_4_, and 0.1 mM PMSF). 30 μg of protein were applied on the SDS-PAGE gel (sodium dodecyl sulfate polyacrylamide) 7.5%. Then, proteins were transferred to a PVDF membrane (Millipore, United States) and the membranes were blocked (3% albumin in TBS enriched with 0.1% Tween 20) before overnight incubation with the specific primary antibody: anti-eNOS (1:750; mouse monoclonal; Santa Cruz Biotechnology Inc., Santa Cruz, CA, United States), anti β-actin (1:1000, mouse monoclonal; Santa Cruz Biotechnology Inc.), anti-phospho-eNOS-Ser^1177^ (1:500; goat polyclonal; Santa Cruz Biotechnology Inc.), and anti-phospho-eNOS-Thr^495^ (1:500, goat polyclonal; Santa Cruz Biotechnology Inc.). The immunocomplexes were detected by a chemiluminescence assay (ECL Plus kit; Amersham, Les Ulis, France) and the densitometry analyses were done using the software ImageJ 1.48v.

### Systolic Blood Pressure Measurement

The systolic blood pressure (SBP) was measured by the tail-cuff plethysmography (Gross and Luft, 2003) using MRBP system (IITC Life Science, Los Angeles, CA, United States). Conscious mice were conditioned in restraints in a warming chamber controlled at 32°C for 10 min. The measurements were taken at 10, 30, and 60 min after the intraperitoneal administration of D-pinitol (10 mg/kg) or saline. The basal SBP was measured 10 min before the administration of D-pinitol.

### Statistical Analysis

Statistical analyses were performed using GraphPad Prism 4 program. Vasodilator data were represented as the percentage of reduction in the sustained contraction induced phenylephrine (3 μM). Two-way ANOVA followed by Bonferroni post-test was used to analyze the cumulative concentration-response curves and the reduction in the SBP induced by D-pinitol. One-way ANOVA was used for the statistical analysis of nitrite dosage and Western blot data. All results were expressed as mean ± standard error of the mean (SEM) and found to be significant if *P* < 0.05.

## Results

### Vascular Reactivity

**FIGURE 1:**
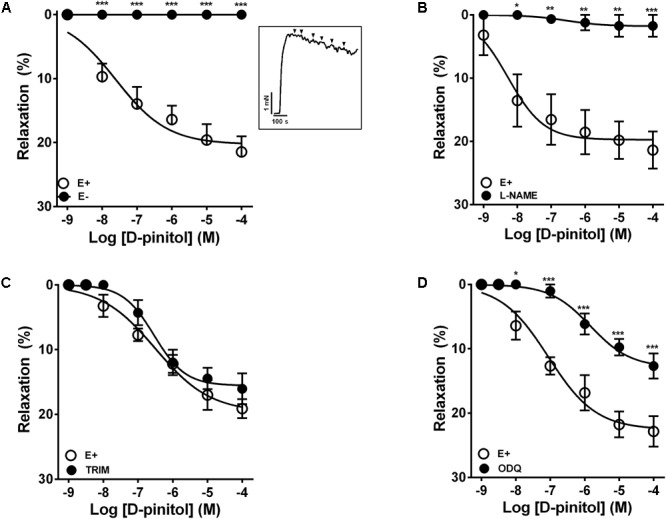
Endothelium- and nitric oxide-dependent vasodilator effect of D-pinitol in mice mesenteric artery. The concentration-dependent vasodilator effect of D-pinitol was investigated in the presence (E+) or absence (E-) of a functional endothelium **(A)**, in the presence of L-NAME **(B)**, TRIM **(C)**, and ODQ **(D)**. The inset demonstrates a representative trace of the concentration-dependent vasodilator effect of D-pinitol, where each mark represent a concentration. All results are expressed as mean ± SEM of five experiments. ^∗^*P* < 0.05, ^∗∗^*P* < 0.01, and ^∗∗∗^*P* < 0.001 versus E+.

**FIGURE 2:**
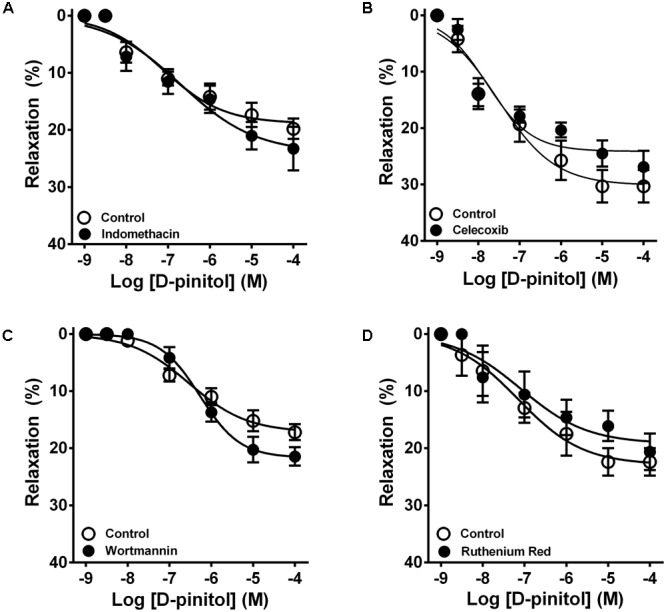
Cyclooxygenase 1 and 2, phosphatidylinositol 3-kinase, and transient receptor potential channels are not involved in the vasodilator effect of D-pinitol in mice mesenteric arteries. The concentration-dependent vasodilator effect of D-pinitol was investigated in mesenteric arteries with a functional endothelium in the absence (Control) and presence of indomethacin **(A)**, celecoxib **(B)**, wortmannin **(C)**, and ruthenium red **(D)**. All results are expressed as mean ± SEM of five experiments.

**FIGURE 3:**
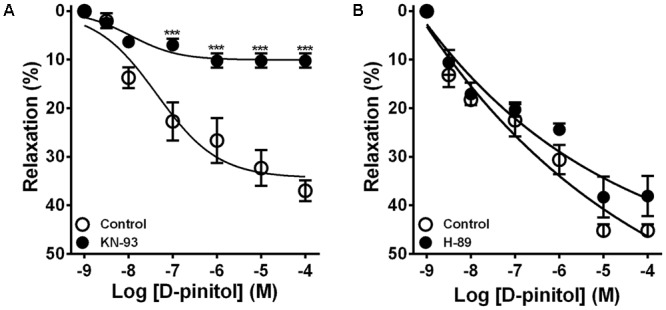
Ca^2+^/calmodulin-dependent kinase II, but not protein kinase A, is involved in the vasodilator effect of D-pinitol in mice mesenteric arteries. The concentration-dependent vasodilator effect of D-pinitol was investigated in mesenteric arteries with a functional endothelium in the absence (control) and presence of KN-93 **(A)** and H-89 **(B)**. All results are expressed as mean ± SEM of five experiments. ^∗∗∗^*P* < 0.001 versus respective control.

**FIGURE 4:**
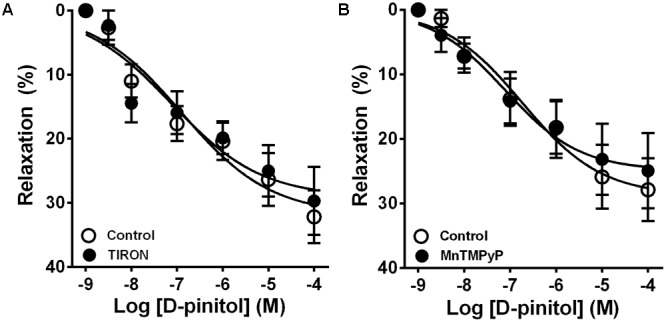
The vasodilator effect of D-pinitol in mice mesenteric arteries is not associated with the formation of reactive oxygen species. The concentration-dependent vasodilator effect of D-pinitol was investigated in mesenteric arteries with a functional endothelium in the absence (control) and presence of tiron **(A)** or MnTMPyP **(B)**. All results are expressed as mean ± SEM of five experiments.

In the presence of a functional endothelium, D-pinitol induced a concentration-dependent vasodilator effect. The maximal vasodilator effect (E_max_) achieved was 21.4 ± 2.4% (**Figure 1A**). However, in the absence of a functional endothelium, the vasodilator effect of D-pinitol was abolished (**Figure 1A**). The non-selective inhibition of eNOS with L-NAME blunted the vasodilator effect of D-pinitol (**Figure 1B**) while the selective inhibition of nNOS with TRIM did not alter its vasodilator effect (**Figure 1C**). The inhibition of guanylate cyclase with ODQ induced a significant shift to the right on the concentration-response curve of D-pinitol (**Figure 1D**). The inhibition of COX-1 with indomethacin (**Figure 2A**), COX-2 with celecoxib (**Figure 2B**), PI3K with wortmannin (**Figure 2C**), and non-selective calcium channels blockade with ruthenium red (**Figure 2D**) did not change the vasodilator effect of D-pinitol. KN-93, a selective inhibitor of CaMKII, blunted the vasodilator effect of D-pinitol (**Figure 3A**), while H-89, a selective inhibitor of PKA, did not change it (**Figure 3B**). Antioxidant drugs such as Tiron (**Figure 4A**) and MnTMPyP (**Figure 4B**), a cell-permeable analog of SOD, did not alter the concentration-response curve to D-pinitol in endothelium-intact mesenteric arteries. Preincubation of arteries with atropine, MPP, and PHTPP did not change the concentration-response curve to D-pinitol (Supplementary Figure S1).

### Nitrite Measurement in Mouse Mesenteric Artery

**FIGURE 5:**
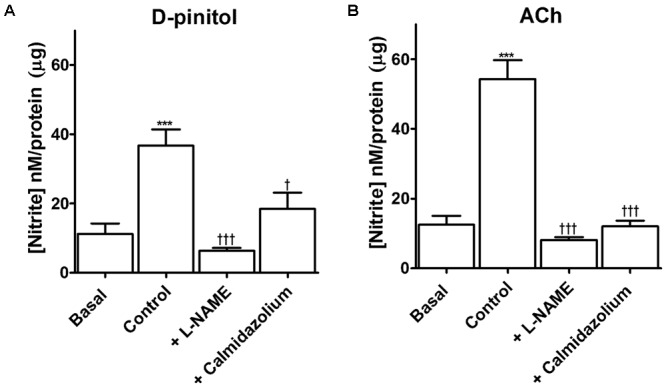
D-pinitol increases the formation of nitrite through a mechanism dependent on the activation of endothelial nitric oxide synthase (eNOS) by the calcium-calmodulin complex. The formation of nitrite was measured in endothelium-intact mesenteric arteries in the absence (basal) or presence of D-pinitol (control/ **A**) or acetylcholine (ACh; control; **B**). The involvement of eNOS and calmodulin was evaluated in mesenteric arteries stimulated with D-pinitol or ACh in the presence of L-NAME and calmidazolium, respectively. All results are expressed as mean ± SEM of five experiments. ^∗∗∗^*P* < 0.001 versus basal. ^†^*P* < 0.05, and ^†††^*P* < 0.001 versus control.

D-pinitol increased more than threefold the amount of nitrite in comparison with the basal level in mouse mesenteric artery (**Figure 5A**). L-NAME blunted the increase induced by D-pinitol while calmidazolium induced a significant reduction (**Figure 5A**). A similar effect was observed with ACh (10 μM), which was also inhibited by L-NAME and calmidazolium (**Figure 5B**).

### Western Blot Analysis of eNOS Phosphorylation

**FIGURE 6:**
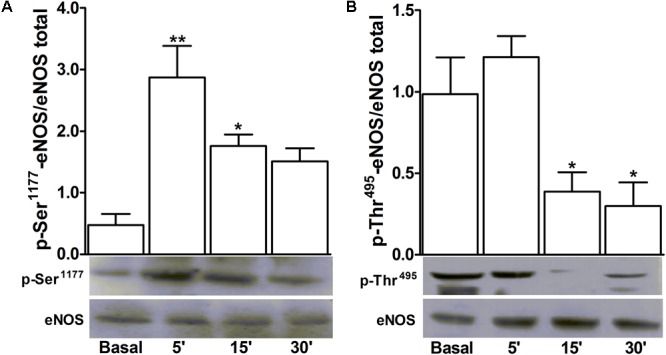
D-pinitol activates the endothelial nitric oxide synthase (eNOS) phosphorylating and dephosphorylating its respective activation and inactivation sites. Western-blots for time-course of the phosphorylation level of Ser1177 **(A)**, the activation site of eNOS, and the dephosphorylation level of Thr495 **(B)**, the inactivation site of eNOS, were performed in endothelium-intact mesenteric arteries, in the absence (basal) and presence of D-pinitol (20 μM). All results are expressed as mean ± SEM of five experiments. ^∗^*P* < 0.05 and ^∗∗^*P* < 0.01 versus basal.

D-pinitol increased the phosphorylation level of the activation site of eNOS at Ser^1177^ 5 and 15 min after the stimulation of endothelium-intact mesenteric arteries (**Figure 6A**). Besides, the level of phosphorylation of the inactivation site of eNOS at Thr^495^ was decreased 15 and 30 min after the stimulation of the arteries with D-pinitol (**Figure 6B**).

### Systolic Blood Pressure Measurement

**FIGURE 7:**
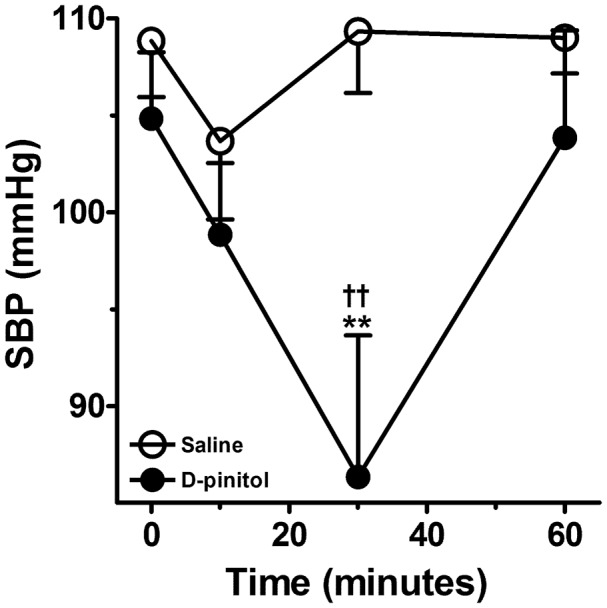
D-pinitol (10 mg/kg; i.p.) reduces the systolic blood pressure (SBP) in normotensive mice. All results are expressed as mean ± SEM of six experiments. ^∗∗^*P* < 0.01 versus saline at 30 min. ^††^*P* < 0.01 versus the SBP measured before the administration of D-pinitol.

D-pinitol induced a significant reduction in the SBP of normotensive mice (**Figure 7**). The significant reduction in the SBP was observed 30 min after the administration of D-pinitol in comparison to the saline SBP at 30 min and to the SBP observed before the administration of D-pinitol.

## Discussion

D-pinitol has been extensively investigated for the treatment of metabolic diseases in humans, as food supplement, and demonstrated protective effects in the cardiovascular system. The present study demonstrated that D-pinitol induces a vasodilator effect in mice mesenteric arteries by a mechanism dependent on the production of endothelium-derived NO through the activation of the calcium-calmodulin complex.

Previous studies with D-pinitol have demonstrated that this cyclitol has vascular endothelium protective effects (Nascimento et al., 2006). The present work described a new biological effect of D-pinitol as a vasodilator drug and contributed to the understanding of its mechanism of action. Although 20–30% of vasodilator effect seems to be relatively small if compared with ACh, this level of effect is compatible with physiological vasodilator mediators, such as angiotensin (1–7) (Neves et al., 2003; Lemos et al., 2005) and substance P (Beny and Brunet, 1988) that play an important role in the control of vascular tone and blood pressure. It is noteworthy remember that the vascular resistance is inversely proportional to the radius of the vasculature at the fourth potency, meaning that a small change in the diameter of the artery results in a significant reduction in the vascular resistance. This is relevant since this cyclitol induced a significant vasodilator effect at concentrations as low as 10 nM and has been tested for the treatment of different pathologies, notably diabetes, at doses as high as 1,200 mg/day by the oral route, reaching plasma concentration above 1 μM (Campbell et al., 2004; Kim et al., 2005; Kang et al., 2006). Therefore, it is plausible to think that the concentrations used in this study are achieved in the systemic circulation during chronic treatment with therapeutic doses of D-pinitol.

The endothelium plays an important role in the cardiovascular system. It functions as a semipermeable barrier between the blood and the vessel wall, regulating vital functions such as vascular tone, response to inflammatory stimuli, and blood coagulation (Triggle et al., 2003; Aird, 2007; Otsuka et al., 2012). The control of vascular tone by the endothelium is due to the synthesis and release of mediators such as NO, prostacyclin, and other endothelium-derived relaxant factors (EDRFs). Some of these EDRFs induce endothelium-derived hyperpolarization (EDH) of vascular smooth muscle, such as H_2_O_2_ and epoxyeicosatrienoic acids (Medhora et al., 2001; Matoba et al., 2002; Chadha et al., 2011). In this study, removal of the endothelium abolished the vasodilator effect of D-pinitol, demonstrating the involvement of the EDRFs in the vasodilator effect of this drug. The involvement of eNOS in the formation of EDRF participating in the control of the vascular tone is already well accepted. However, several studies have demonstrated the presence of nNOS in the endothelium of mice mesenteric arteries and aorta, validating its participation in the control of the vascular tone (Capettini et al., 2008, 2010; Takaki et al., 2008; Silva et al., 2016). The inhibition of the vasodilator effect of D-pinitol by L-NAME and the absence of inhibitory effect by TRIM, suggest the endothelium-dependent effect of D-pinitol occurs through the activation of eNOS, and that nNOS is not involved. The reduction of the vasodilator effect of D-pinitol by ODQ, a selective inhibitor of guanylate cyclase, supports the participation NO as the eNOS-derived EDRF in the vasodilator effect of this cyclitol in mice mesenteric arteries. In addition, the absence of effect of COX inhibitors rules out the participation of prostacyclin in the vasodilator effect of D-pinitol.

eNOS can be activated by calcium-independent and -dependent mechanisms (Fleming, 2010). The calcium-independent mechanism of the activation of eNOS involves the PI3K/Akt pathway (Dimmeler et al., 1999; Fulton et al., 1999). Wortmannin, a selective inhibitor of PI3K (Arcaro and Wymann, 1993), prevents the phosphorylation and activation of Akt and inhibits increased levels of cGMP in vascular smooth muscle (Dimmeler et al., 1999). In the present work, wortmannin did not change the vasodilator effect of D-pinitol, suggesting that the PI3K/Akt pathway is not involved in the activation of eNOS by this cyclitol in mesenteric arteries. The calcium-dependent mechanism involves the increase in intracellular calcium concentration resulting from the release of intracellular stores of calcium or the influx through the plasmatic membrane of endothelial cells (Fleming, 2010; Forstermann and Sessa, 2012). The transient receptor potential channels (TRPs) are considered non-selective channels that allow the influx of calcium, sodium, and magnesium (Pedersen et al., 2005; Owsianik et al., 2006). In the endothelial cells the vanilloid TRPs (TRPV), inhibited by ruthenium red, have been described as the main TRPs involved in the endothelium-dependent vasodilatation in mouse mesenteric artery (Zhang et al., 2009). The absence of a significant modification in the concentration-response curve of D-pinitol in the presence of ruthenium red suggests that the TRPVs are not involved in the vasodilatation induced by this cyclitol. Thus, the calcium-dependent mechanism of eNOS activation induced by D-pinitol can be related to the release of the intracellular calcium stores or by the influx of calcium through other channels. The calcium-dependent mechanism occurs through the formation of calcium-calmodulin complex (Matsubara et al., 1996; Fleming, 2010). This mechanism is inhibited by calmidazolium, a calmodulin antagonist (Busse and Mulsch, 1990). In the present work, the effect of calmidazolium was not investigated in the vasodilator effect of D-pinitol as this drug inhibited the contraction induced by phenylephrine. However, calmidazolium significantly inhibited the production of nitrite induced by D-pinitol, in a similar way as observed with ACh. Therefore, D-pinitol seems to activate eNOS by a calcium-dependent mechanism.

The phosphorylation of Ser^1177^ is associated with the activation of eNOS (Michel et al., 1993; Gallis et al., 1999). Kinases such as protein kinase B (PKB), adenosine monophosphate-activated protein kinase (AMPK), protein kinase A (PKA) and CaMKII can be involved in the activation of eNOS (Schneider et al., 2003; Fisslthaler and Fleming, 2009; Forstermann and Sessa, 2012). The absence of inhibitory effect for H-89 demonstrates that PKA is not involved in the vasodilator effect of D-pinitol. The inhibition of the vasodilator effect of D-pinitol by KN-93 confirms the participation of CaMKII in the activation of eNOS and supports a calcium-dependent mechanism for D-pinitol. In order to confirm the activation of eNOS, the level of phosphorylation of Ser^1177^ was investigated in the presence of D-pinitol by Western blot. The significant increase in the level of phosphorylation of Ser^1177^ at 5 and 15 min after the stimulation with D-pinitol confirms the ability of this cyclitol to activate eNOS. Additionally, the activity of eNOS is also regulated by inactivation sites, such as Thr^495^ (Fleming et al., 2001). Substances with the ability to activate eNOS reduce the phosphorylation of this site. As observed by Western blot the stimulation with D-pinitol was able to reduce the level of phosphorylation of Thr495 significantly, once more confirming the ability of this cyclitol to activate eNOS. This is an important point since Nascimento et al. (2006) suggested that the mechanism involved in the increased bioavailability of NO induced by D-pinitol in arteries from diabetic animals was related to its antioxidant property, while the direct vasodilator effect of D-pinitol was not demonstrated in this previous report. The absence of effect of antioxidant drugs such as tiron and MnTMPyP on the vasodilator effect of D-pinitol, associated to the profile of phosphorylation discussed above, demonstrate that D-pinitol increases the production of NO and induces its vasodilator effect by activation of eNOS rather than by its antioxidant action.

Finally, the present study also demonstrates that D-pinitol is able to reduce the SBP in normotensive mice. This observation is in line with the vasodilator effect of D-pinitol described above, which suggests a significant reduction in the systemic vascular resistance and blood pressure. A previous report demonstrated that the administration of D-pinitol reduced the cardiovascular risk factors in patients with type 2 diabetes mellitus, including the reduction in the SBP and diastolic blood pressure (Kim et al., 2005). These observations suggest that D-pinitol has a significant effect on the cardiovascular system and may be investigated as an antihypertensive drug. However, attention should be taken if D-pinitol is used as food supplement, considering that high amounts of this cyclitol can significantly reduce the blood pressure and may produce hypotension in normotensive subjects.

The present study allows us to conclude that D-pinitol has an endothelium- and NO-dependent vasodilator effect by a mechanism of action involving the calcium-calmodulin complex activation of eNOS, which might be responsible for its hypotensive effect.

## Author Contributions

LM, JS, and GS performed research and contributed to the writing of the manuscript. VL and SC designed research and reviewed the manuscript to the submission version.

## Conflict of Interest Statement

The authors declare that the research was conducted in the absence of any commercial or financial relationships that could be construed as a potential conflict of interest.
