# Supplementary material for: Activation of eNOS by D-pinitol Induces an Endothelium-Dependent Vasodilatation in Mouse Mesenteric Artery
Source: Front Pharmacol. 2018 May 22;9:528. doi: 10.3389/fphar.2018.00528 (PMC5972298; doi:10.3389/fphar.2018.00528)
Supplement: Supplementary file 1 [file Image_1.PDF]

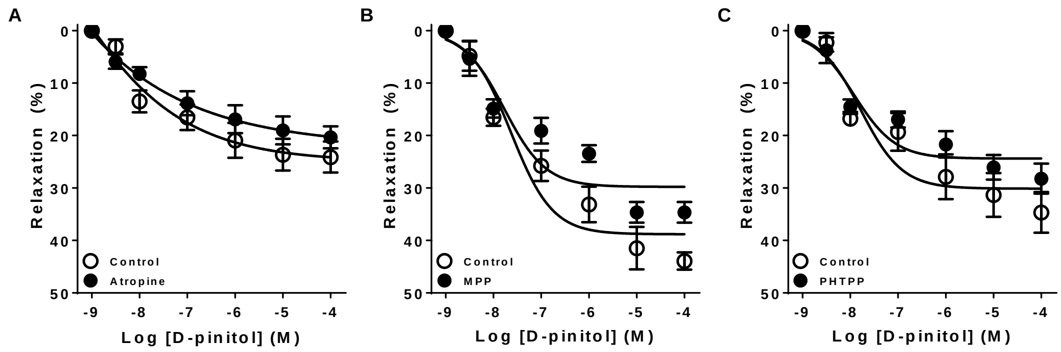

Supplementary Figure 1. Muscarinic receptors are not involved in the vasodilator effect of D-pinitol in mice mesenteric arteries. The concentration-dependent vasodilator effect of D-pinitol was investigated in mesenteric arteries with a functional endothelium in the absence (Control) and presence of atropine (1 µM; **A**), MPP (0.1 µM; **B**), and PHTPP (0.1 µM; **C**). All results are expressed as mean ± SEM of six experiments.
